# Prevalence and Heterogeneity of Swine Influenza Virus in China From 2010 to 2025: A Systematic Review and Meta‐Analysis

**DOI:** 10.1155/tbed/1096796

**Published:** 2026-02-13

**Authors:** Xiutao Yang, Qingxia Gao, Zhaofang Xi, Pengfei Zhao, Junlong Zhao

**Affiliations:** ^1^ National Key Laboratory of Agricultural Microbiology, College of Veterinary Medicine, Huazhong Agricultural University, Wuhan, Hubei, China, hzau.edu.cn; ^2^ Chia Tai Group (Central South) Research Institute, Xiangyang Chia Tai Agro-Industry and Food Co., Ltd., Xiangyang, Hubei, China

**Keywords:** China, epidemiological surveillance, heterogeneity, meta-analysis, prevalence, swine influenza virus

## Abstract

**Background:**

Swine influenza virus (SIV) is endemic in China, threatening the swine industry and public health. This meta‐analysis estimated the national pooled prevalence of SIV (2010–2025) and identified key sources of heterogeneity.

**Methods:**

Following Preferred Reporting Items for Systematic Reviews and Meta‐Analyses (PRISMA) guidelines, six databases were searched, yielding 73 eligible studies with 411,930 samples. A random‐effects model pooled prevalence estimates, and subgroup analyses explored heterogeneity.

**Results:**

The pooled SIV prevalence was 30.3% (95% confidence interval [CI]: 24.5%–36.4%) with extreme heterogeneity (*I* ^2^ = 100%, *p*  < 0.001). Key drivers included diagnostic method (serological: 37.1% vs. virological: 2.6%), geography (Southwest China: 54.3%), and viral genotype (H1 > H3). Sensitivity analysis confirmed robustness, but publication bias (Egger’s test, *p* = 0.0009) suggests potential overestimation.

**Conclusion:**

SIV is widespread in China but exhibits marked spatiotemporal and methodological variability. A single national prevalence figure is insufficient for risk assessment. Surveillance and control strategies must be targeted and context‐specific. This study provides a critical, albeit potentially overestimated, epidemiological baseline for evidence‐based interventions.

## 1. Introduction

Swine influenza virus (SIV), an Orthomyxovirus, *Alphainfluenzavirus* type A, is a primary etiological agent of acute respiratory disease in pigs, imposing a significant economic burden on the global swine industry and posing a considerable public health challenge [1, 2]. China, the world’s largest producer and consumer of pork, presents a unique ecological landscape for SIV evolution. The industry’s characteristics, including large‐scale, high‐density farming operations and frequent interregional animal transport, create ideal conditions for the persistent circulation, mutation, and reassortment of SIV [3]. The introduction and rapid establishment of the pandemic H1N1/09 (pdm09 H1N1) virus profoundly altered the national epidemiology of SIV in Chinese swine herds. This strain has since undergone extensive genetic reassortment with endemic subtypes, such as classical swine H1N1 (CS H1N1), Eurasian avian‐like H1N1 (EA H1N1), and H3N2, which has fueled a rapid increase in viral genetic diversity and driven the continuous emergence of novel reassortant viruses [4, 5].

Existing research has established a significant spatiotemporal heterogeneity in SIV prevalence across China. Seroprevalence is consistently higher in the densely farmed southern and eastern regions compared to the northern and western provinces, and infection rates follow a seasonal pattern, peaking during winter and spring [6]. Furthermore, coinfections involving multiple subtypes are widespread. This phenomenon not only compounds production losses but also elevates the risk of novel reassortant viruses emerging [7]. Given that pigs serve as critical “mixing vessels” in the global influenza ecosystem, a systematic and panoramic quantitative assessment of SIV in China is paramount. Such an analysis is vital for elucidating macroevolutionary viral dynamics, identifying emerging public health threats, and developing evidence‐based control strategies [8].

Since 2010, epidemiological research on SIV in China has advanced through two distinct phases. The initial phase (2010–2016) was characterized by regional serological surveys and basic pathogen characterization, primarily employing enzyme‐linked immunosorbent assay (ELISA) and hemagglutination inhibition (HI) assays. Studies from southern provinces like Guangxi, Hunan, and Guangdong, for example, identified H1N1 as the dominant subtype with high seroprevalence, while H3N2 circulated at lower levels [9–12]. This body of work established a preliminary “south‐high, north‐low” geographic distribution and confirmed that prevalence was dependent on season and age [8, 13]. During the same period, virological surveillance began tracking the incursion and spread of the pdm09 H1N1 virus, identifying highly active genetic reassortment through the discovery of diverse triple‐reassortant viruses in provinces such as Liaoning and Shandong [14, 15]. Since 2017, the focus of SIV research has matured, shifting toward integrated multisubtype surveillance, genomic epidemiology, and quantitative risk assessment. Bolstered by enhanced national surveillance networks and the widespread adoption of molecular technologies, the scope and depth of these studies have grown considerably. Surveillance has expanded to historically under‐sampled regions, including Tibet and Ningxia, which has reinforced the nationwide predominance of the H1N1 subtype [16]. Virological investigations have broadened beyond major swine subtypes to systematically monitor the spillover of avian influenza viruses (e.g., H5, H9) into pig populations and to more accurately quantify co‐infection rates and patterns [17]. Critically, the adoption of advanced analytical tools like phylodynamics has enabled researchers to reconstruct viral evolutionary pathways and trace interregional transmission dynamics. Despite this progress, the existing literature suffers from significant limitations, including inconsistencies in sample sizes, diagnostic methods, and study durations, as well as unbalanced geographic coverage. These shortcomings have precluded the formation of a cohesive, robust national picture of SIV epidemiology. Consequently, a meta‐analysis to systematically synthesize the available evidence is urgently needed.

This study will provide a systematic evaluation of the overall prevalence and key determinants of SIV in China between 2010 and 2025. Our methodology will strictly adhere to the Preferred Reporting Items for Systematic Reviews and Meta‐Analyses (PRISMA) guidelines [18]. A comprehensive literature search will be conducted across English and Chinese databases (PubMed, China National Knowledge Infrastructure [CNKI], Wanfang Data, and Web of Science) to identify all original research meeting our predefined inclusion and exclusion criteria. Two researchers will independently screen articles and extract key data, including bibliographic details, sample demographics, diagnostic methods, and prevalence metrics. By quantitatively synthesizing 15 years of epidemiological data, this research will deliver a comprehensive, objective, and high‐evidence summary of SIV dynamics in China. The findings are intended to pinpoint high‐risk regions, transmission pathways, and viral subtypes, providing a robust scientific foundation for national and local authorities to design differentiated and targeted surveillance and vaccination strategies. Ultimately, by clarifying the epidemiological burden of SIV, this work will empower the swine industry to optimize resource allocation, mitigate economic losses, and foster its own healthy and sustainable development.

## 2. Materials and Methods

### 2.1. Literature Search and Screening Strategy

This systematic review and meta‐analysis was designed and executed in strict adherence to the PRISMA 2020 guidelines [18]. We conducted a systematic search of English and Chinese electronic databases to identify all original research articles reporting the SIV prevalence in China, published between January 1, 2010, and May 31, 2025. The search covered Google Scholar (4080 records), Web of Science (554), PubMed (31), CNKI (1047), Chaoxing Journals Database (205), and Wanfang Data (210), yielding a total of 6127 initial records. The specific search strategies for each database were as follows: (1) Google Scholar: (“swine influenza virus”) AND (“prevalence” OR “seroprevalence” OR “epidemiology”) AND (“China” OR “Chinese”). (2) Web of Science: TS = (“swine influenza virus” OR “porcine influenza virus” OR “swine‐origin influenza virus” OR (“influenza a virus” AND (“pigs” OR “swine” OR “boar” OR “piglet” OR “sow"))) AND TS = (“prevalence” OR “seroprevalence” OR “antigen” OR “molecular prevalence” OR “epidemiology” OR “serosurvey” OR “serosurveillance”) AND TS = (“China” OR “Chinese”). (3) PubMed: ((“swine influenza virus” [tiab] OR “porcine influenza virus” [tiab] OR “swine‐origin influenza virus” [tiab] OR ("influenza a virus” [tiab] AND (“pigs” [tiab] OR “swine” [tiab] OR “boar” [tiab] OR “sow” [tiab] OR “piglet” [tiab]))) AND (“prevalence” [tiab] OR “seroprevalence” [tiab] OR “antigen” [tiab] OR “molecular prevalence” [tiab] OR “epidemiology” [tiab] OR “serosurvey” [tiab] OR “serosurveillance” [tiab]) AND (“China” [tiab] OR “Chinese” [tiab])). (4) CNKI (in Chinese): T = (“swine flu” OR “swine flu virus” OR “swine influenza” OR “H1N1 swine flu” OR “swine flu infection” OR “swine flu pathogen") AND T = (“prevalence” OR “infection rate” OR “serological positivity” OR “antigen positivity” OR “molecular prevalence” OR “morbidity” OR “epidemiological survey” OR “serological survey”) AND T = (“China” OR “domestic” OR “mainland China” OR “mainland China” OR “provinces in China” OR “Chinese farms”). (5) Super Star Journal Database (in Chinese): (“swine flu” OR “swine flu virus”) AND (“prevalence” OR “epidemiological survey”) AND (“China” OR “domestic”). (6) Wanfang Data (in Chinese): SU = (“swine flu” + “swine flu virus”) _SU = (“China” + “domestic” + “mainland China”) _SU = (“prevalence” + “infection rate” + “morbidity” + “serological survey” + “surveillance data”). The search was conducted without language restrictions. All retrieved citations were imported into EndNote X9 (Clarivate Analytics, Philadelphia, PA, USA) for management and deduplication. Following this, two reviewers independently screened the titles and abstracts against pre‐specified eligibility criteria. Discrepancies were resolved by discussion or, if necessary, adjudicated by a third expert. Initial exclusion criteria were (1) topic irrelevant to SIV prevalence; (2) study population outside of mainland China; (3) nonoriginal article format (e.g., review, editorial, and conference abstract); (4) etiological agent other than SIV; or (5) ineligible publication type (e.g., book chapter). During the full‐text assessment, articles were excluded for the following reasons: (1) redundant publication of the same dataset (*n* = 5); (2) the epidemiological unit was the farm rather than the individual animal (*n* = 2); (3) study objectives were incongruent with this meta‐analysis (*n* = 12); (4) sampling occurred before 2010 (*n* = 9); (5) inadequate sample size (*n* = 21), defined as fewer than 427 individuals. Although meta‐analyses typically encompass studies of diverse scales, this threshold was rigorously applied to ensure that each included primary study possessed the requisite statistical power. Specifically, the criterion guaranteed a prevalence estimation with a margin of error not exceeding 5%, predicated on a conservative prevalence of 50% and a 95% confidence interval (CI). This stringent quality control measure was implemented to mitigate the influence of small‐scale, opportunistic studies, which are frequently prone to sampling bias and resultant extreme estimates [19]; (6) subjects were human (*n* = 3); (7) the pathogen of interest was avian influenza virus (*n* = 4); or (8) the full text was unobtainable (*n* = 1).

### 2.2. Data Extraction and Quality Appraisal

Using a standardized data extraction instrument developed in Microsoft Excel 2019 (Microsoft Corporation, Redmond, WA, USA), two reviewers independently extracted relevant information from each included study. All extracted data were subsequently cross‐verified for accuracy. Key extracted variables included first author, publication year, geographical location (province), swine production stage (e.g., gilt, suckling piglet, weaned pig, finishing pig, and boar), diagnostic method (e.g., ELISA, HI, and real‐time reverse transcription polymerase chain reaction [RT‐PCR], and virus isolation), SIV subtype/genotype, number of positive samples, and total sample size. Data were then systematically categorized for subgroup analyses based on geographical region (seven regions), province, production stage, diagnostic method, viral genotype, publication period (2010–2015, 2016–2020, 2021–2025), and study quality. All discrepancies encountered during this process were resolved through consensus. The methodological quality of each study was appraised using a modified Newcastle–Ottawa Scale (NOS) adapted for cross‐sectional study designs [20]. This 10‐point scale evaluates domains such as study design transparency, sample representativeness, methodological standardization, data completeness, and the control of confounding variables (see Table 1 for the full rubric). Based on their total score, studies were categorized as low (0–4 points), medium (5–7 points), or high quality (8–10 points). These quality ratings were incorporated into subsequent analyses to assess their potential influence on the pooled prevalence estimates.

**Table 1:** Quality assessment scale for studies on SIV epidemiology.

Item	Assessment domain	Scoring criteria	Rationale
1	Transparency of study design	2 points: Study type (eg., cross‐sectional) is explicitly stated with a detailed description of the implementation process. 1 point: Study type is mentioned, but details of the design are insufficient. 0 points: Study design is not described	A clear study design is fundamental for assessing the internal validity and reliability of the research findings
2	Representativeness of the sample	2 points: The sample covers multiple geographical regions or was selected using a probability sampling method (eg., random sampling), ensuring high representativeness. 1 point: The sample is from a single or few regions using nonprobability sampling but is still reasonably representative of the target population. 0 points: The sample source is singular, the sampling method is unclear, or representativeness is insufficient/cannot be judged	The representativeness of the sample directly determines the generalizability of the study’s findings to the broader target swine population
3	Standardization and description of detection method	2 points: A standardized method (eg., RT‐PCR, ELISA, and HI) was used, with detailed operating parameters or citation of a standard protocol. 1 point: The name of the detection method is mentioned, but key operational details or quality control measures are lacking. 0 points: The detection method is not clearly described, or a nonstandardized method was used	The reliability and standardization of the detection method are critical for ensuring data accuracy and inter‐study comparability
4	Completeness of key data reporting	2 points: Core data such as total sample size, number of positive samples, sampling time, and sampling location are clearly reported. 1 point: Some core data are reported, but minor information (eg., specific sampling date range) is missing. 0 points: Key data essential for calculating prevalence (eg., number of positives or total sample size) are missing	The completeness of data is a prerequisite for the quantitative synthesis required in a meta‐analysis
5	Control of confounding factors	2 points: One or more important potential confounders (eg., pig age group, season, and vaccination history) were controlled for in the analysis through stratification or statistical adjustment. 1 point: Potential confounders were collected or discussed but not effectively controlled for in the analysis. 0 points: No potential confounding factors were considered or mentioned	Effective control of confounders helps to provide a more accurate estimate of the true SIV prevalence and reduces bias

### 2.3. Statistical Analysis

We pooled the prevalence estimates from the included studies using the metaprop function of the meta package in R [21]. Raw prevalence data were transformed using the Freeman–Tukey double arcsine method to stabilize variances, a necessary step for proportions approaching 0 or 1 [22]. A random‐effects model was chosen a priori to account for the expected substantial heterogeneity across studies arising from variations in geography, time, and methodology. The between‐study variance (*τ* ^2^) was estimated using the restricted maximum likelihood (REML) approach [23]. Statistical heterogeneity was quantified using the *I* ^2^ statistic, where values of <25%, 25%–50%, and ≥50% were interpreted as low, moderate, and high heterogeneity, respectively, and assessed for significance using Cochran’s *Q* test [24]. We performed a leave‐one‐out sensitivity analysis to evaluate the influence of each individual study on the overall pooled estimate [25, 26]. Publication bias was assessed visually via funnel plots and formally tested with Egger’s regression analysis for subgroups containing 10 or more studies [21, 27]. To explore sources of heterogeneity, we conducted subgroup analyses based on pre‐specified study characteristics (geographical region, province, pig age, detection method, SIV genotype, time period, and study quality). These analyses were implemented using a generalized linear mixed model (GLMM) with a logit link function [28], applying the Hartung–Knapp adjustment to CIs and calculating prediction intervals to reflect the range of true effects [29]. All statistical analyses were performed in R v4.3.1 [30], utilizing the meta, metafor, ggplot2, and dplyr packages. A two‐sided *p*‐value <0.05 was considered statistically significant for all tests.

## 3. Results

### 3.1. Study Selection and Characteristics

**Figure 1:**
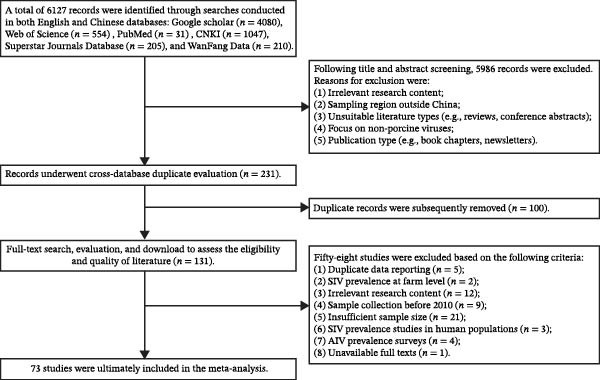
PRISMA flow diagram of the literature search and study selection for the meta‐analysis on the prevalence of SIV in China from 2010 to 2025.

Our systematic search of six databases yielded 6127 records. Following the PRISMA workflow (Figure 1), 131 articles remained for full‐text assessment after duplicate removal and initial screening. A final cohort of 73 studies, which met all eligibility criteria, was included in the quantitative synthesis. The characteristics of these 73 studies are detailed in Table 2 and Table S1. Collectively, they represent a substantial dataset of 411,930 individual porcine samples from across China, of which 100,135 were positive for SIV (crude overall prevalence: 24.31%). The reported prevalence across studies was highly variable, ranging from a low of 0.14% [80] to a high of 81.76% [73], foreshadowing significant heterogeneity. Methodological quality, assessed via a modified NOS scale, was predominantly moderate to high: 7 studies (9.6%) were rated as high quality, 62 (84.9%) as medium quality, and only 4 (5.5%) as low quality. This indicates a solid foundation for the meta‐analysis.

**Table 2:** Studies included in the meta‐analysis of SIV prevalence in China from 2010 to 2025.

Number	Author	Total size	Positive size	Prevalence (%)	Grade
1	Anderson et al. [31]	3888	268	6.89	Medium
2	Cai et al. [32]	2611	640	24.51	Medium
3	Cao et al. [33]	1050	529	50.38	Medium
4	Chen et al. [34]	1170	290	24.79	Medium
5	Huang et al. [11]	2540	192	7.56	Medium
6	Lan et al. [35]	2161	749	34.66	Medium
7	Li et al. [8]	35,738	1605	4.49	Medium
8	Liang et al. [36]	758	186	24.54	High
9	Liu et al. [37]	5856	3368	57.51	Medium
10	Sui et al. [38]	5852	2932	50.10	Medium
11	Sun et al. [39]	4193	45	1.07	Medium
12	Wang et al. [40]	874	642	73.46	Medium
13	Yin et al. [41]	13,044	5748	44.07	Medium
14	Zhang et al. [42]	545	50	9.17	Medium
15	Zhou et al. [43]	1180	219	18.56	Low
16	He et al. [44]	1796	1061	59.08	Medium
17	Zhao et al. [45]	40,343	19,980	49.53	High
18	Gan et al. [46]	2005	978	48.78	Medium
19	Meng et al. [47]	103,110	855	0.83	Medium
20	Sun et al. [48]	995	651	65.43	Medium
21	Zhang et al. [49]	475	16	3.37	Low
22	Zhao et al. [50]	2972	24	0.81	Medium
23	Fu et al. [51]	3265	2058	63.03	Medium
24	Ren et al. [52]	500	6	1.20	Medium
25	Wu et al. [53]	2808	1269	45.19	Medium
26	Cai et al. [54]	15,241	3473	22.79	Medium
27	Chen et al. [55]	779	490	62.90	Medium
28	Chen et al. [56]	937	411	43.86	Medium
29	Yu et al. [57]	758	338	44.59	Medium
30	Huang et al. [58]	1573	524	33.31	Medium
31	Li et al. [59]	3991	950	23.80	Medium
32	Li et al. [60]	920	358	38.91	Medium
33	Ma et al. [61]	1200	456	38.00	Medium
34	Oyang et al. [62]	873	501	57.39	Medium
35	Wang et al. [63]	1519	553	36.41	Medium
36	Wei et al. [64]	572	304	53.15	Medium
37	Xu et al. [65]	1015	798	78.62	Medium
38	Xu et al. [66]	729	205	28.12	Medium
39	Yao et al. [67]	1173	14	1.19	Medium
40	Yu et al. [68]	17,861	424	2.37	Medium
41	Zhang et al. [69]	498	180	36.14	Low
42	Cai et al. [70]	1014	221	21.79	Medium
43	Cao [71]	10,188	4413	43.32	High
44	Cui [72]	1840	297	16.14	Medium
45	Zhai et al. [73]	877	717	81.76	Medium
46	Fang [74]	3644	2040	55.98	Medium
47	Han [75]	17,453	6852	39.26	High
48	Hu et al. [76]	5514	3105	56.31	Medium
49	Huang [77]	12,364	216	1.75	Medium
50	Huang et al. [78]	2436	983	40.35	Medium
51	Jin et al. [79]	1760	320	18.18	Medium
52	Ju et al. [80]	720	1	0.14	Low
53	Liu et al. [81]	1210	405	33.47	Medium
54	Liu [82]	914	291	31.84	Medium
55	Lu [83]	545	50	9.17	Medium
56	Luo et al. [84]	784	48	6.12	Medium
57	Shi et al. [85]	1074	768	71.51	Medium
58	Wang [86]	2284	961	42.08	High
59	Wang et al. [87]	1710	211	12.34	Medium
60	Wang [88]	7000	4465	63.79	Medium
61	Xie et al. [89]	3694	1008	27.29	Medium
62	Yu [90]	2127	92	4.33	Medium
63	Zhao [91]	17,865	9315	52.14	High
64	Zhong [92]	4920	2241	45.55	High
65	Zhu [93]	1581	171	10.82	Medium
66	Zhu [94]	1973	162	8.21	Medium
67	Gao [95]	4800	2145	44.69	Medium
68	Liang [96]	758	44	5.80	Medium
69	Lin et al. [97]	1371	359	26.19	Medium
70	Luo et al. [98]	605	454	75.04	Medium
71	Qin [99]	2744	1495	54.48	Medium
72	Wang et al. [100]	1169	637	54.49	Medium
73	Xie et al. [101]	5624	1308	23.26	Medium
Total	—	411,930	100,135	24.31	—

### 3.2. Pooled Prevalence Estimate and Heterogeneity

**Figure 2:**
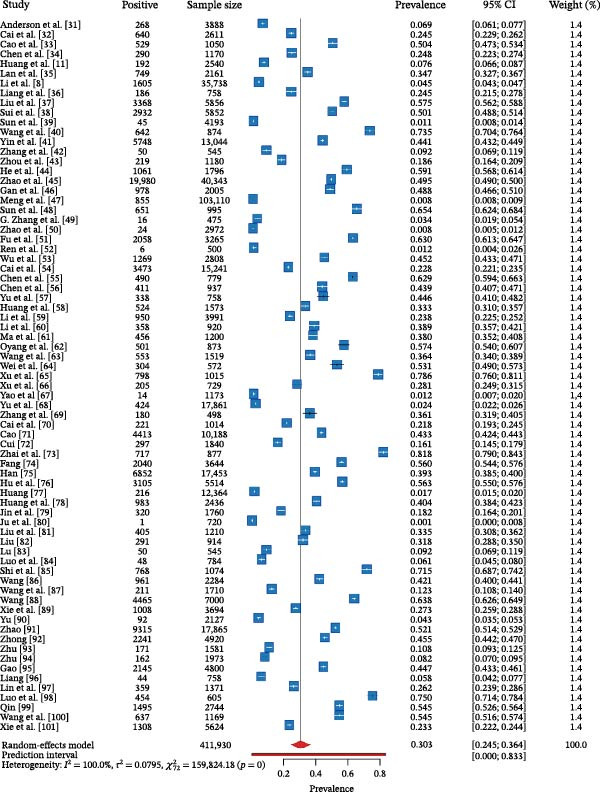
Forest plot of the pooled prevalence of SIV in pigs in China from 2010 to 2025. Note: Each blue square represents the point estimate of prevalence from an individual study, with the size of the square being proportional to the weight assigned to that study in the random‐effects model. The horizontal lines passing through the squares indicate the 95% CI for each study. The red diamond at the bottom represents the pooled prevalence estimate from all studies, and the width of the diamond corresponds to its 95% CI. The red solid line at the very bottom indicates the 95% prediction interval, which estimates the potential range of true prevalence in a future study. Heterogeneity statistics (*I* ^2^, *τ* ^2^) and the *Q*‐statistic are presented at the bottom of the plot.

Employing a random‐effects model, the pooled prevalence of SIV across all 73 studies was estimated to be 30.3% (95% CI: 24.5%–36.4%) for the period of 2010–2025 (Figure 2). However, the analysis revealed extreme and statistically significant heterogeneity among studies (Cochran’s *Q* = 159,824.18, *p* < 0.001; *I* ^2^ = 100%; *τ* ^2^ = 0.0795). The *I* ^2^ value of 100% indicates that the observed variability in prevalence is almost entirely due to true differences between studies (e.g., in methodology, location, or population) rather than random sampling error. Reflecting this immense heterogeneity, the 95% prediction interval was exceptionally wide, spanning from 0.00% to 83.3%. This interval suggests that while our best estimate for the average prevalence is 30.3%, the true prevalence in any single future study could fall anywhere within this vast range, underscoring the profound diversity of SIV epidemiology in China.

### 3.3. Subgroup Analyses of SIV Prevalence

**Figure 3:**
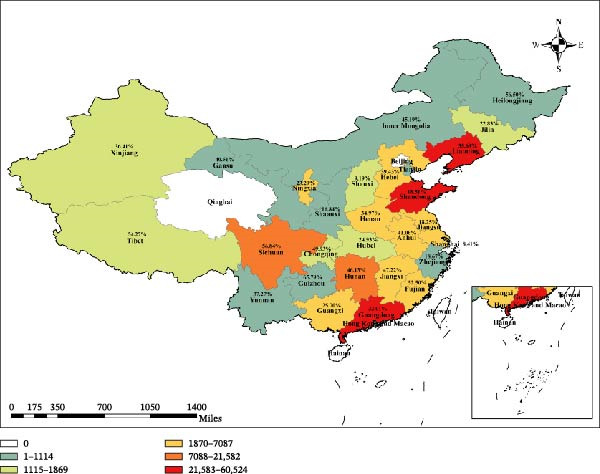
Geographical distribution of sample sizes from Chinese provinces included in the meta‐analysis. The color scale indicates the number of porcine samples collected from each province for the studies included in this meta‐analysis states.

To investigate the sources of the profound heterogeneity, we conducted a series of pre‐specified subgroup analyses (Table 3). The geographic distribution of samples was wide, covering 27 provinces, but was concentrated in major swine‐producing regions like Guangdong, Shandong, and Liaoning (Figure 3). Geographical region was a significant moderator of prevalence (*p* = 0.0238). The pooled prevalence was highest in Southwest China (54.26%; 95% CI: 41.40%–66.58%) and lowest in Northwest China (25.66%; 95% CI: 13.07%–44.20%). Provincial estimates varied even more widely (Table 4). Diagnostic modality was a primary driver of heterogeneity (*p* < 0.0001). As expected, serological assays, which detect past exposure, yielded much higher prevalence estimates (ELISA: 37.09%; HI: 32.85%) than methods that detect active viral presence (RT‐PCR: 2.55%; virus isolation: 0.85%). Viral genotype also significantly influenced prevalence (*p* < 0.0001); H1 subtypes were consistently more prevalent (e.g., EA H1N1 at 26.59%) than H3 subtypes (e.g., H3N2 at 3.58%). Stratification by time period revealed a prevalence of 27.28% (2010–2015), 32.14% (2016–2020), and 9.04% (2021–2025), though the overall temporal trend did not reach statistical significance (*p* = 0.1344). Methodological quality was a significant factor (*p* = 0.0009), with high‐quality studies yielding a significantly higher pooled estimate (42.13%) than medium‐ (25.25%) or low‐quality (5.00%) studies. While prevalence was highest in gilts (43.12%) and boars (41.24%), differences between pig age/production groups were not statistically significant (*p* = 0.2384). Collectively, these analyses identify geographical location, diagnostic approach, viral genotype, and study quality as key sources of the observed heterogeneity.

**Table 3:** Subgroup analysis of SIV prevalence in China (2010–2025).

Variable	Category	No. of studies	No. of sample	No. of positive	Prevalence with 95% CI (%)	Heterogeneity (*χ* ^2^)	*I* ^2^ (%)	*p* Value
Region	Central China	17	24,186	3106	36.60 (19.14–58.47)	5019.66	99.7	0.0238 ^∗^
	Eastern China	31	73,799	30,895	28.16 (17.77–41.57)	5654.40	99.5	—
	North China	12	5779	1883	32.00 (17.77–50.62)	462.72	97.6	—
	Northeast of China	10	49,528	7529	33.69 (17.61–54.71)	8961.50	99.9	—
	Northwest of China	10	13,472	3695	25.66 (13.07–44.20)	483.22	98.1	—
	South China	33	67,611	19,690	30.20 (21.37–40.78)	5654.40	99.5	—
	Southwest of China	14	25,304	11,650	54.26 (41.40–66.58)	1861.66	99.3	—
Detection method	ELISA	29	42,264	17,027	37.09 (26.96–48.50)	3799.45	99.3	<0.0001 ^∗∗∗^
	HI	40	209,771	81,919	32.85 (24.33–42.66)	20352.64	99.8	—
	RT‐PCR	18	77,753	1874	2.55 (1.00–6.37)	1956.66	99.1	—
	Virus isolation	11	128,761	1051	0.85 (0.59–1.23)	42.08	76.2	—
Genotype	CS H1N1	9	40,128	6952	12.20 (5.91–23.52)	1739.46	99.5	<0.0001 ^∗∗∗^
	EA H1N1	23	256,931	50,185	26.59 (18.70–36.32)	23223.37	99.9	—
	Pdm09 H1N1	20	149,293	23,779	18.76 (15.09–23.07)	6646.39	99.7	—
	H1N1	20	48,600	9914	14.61 (6.46–29.76)	4037.97	99.5	—
	H1N2	3	13,190	55	0.43 (0.00–71.15)	129.32	98.5	—
	H3N2	31	113,054	8161	3.58 (1.45–8.57)	6591.00	99.5	—
	H1	17	17,791	6102	33.87 (23.92–45.49)	1558.42	99.0	—
	H3	17	31,570	6572	19.32 (10.27–33.37)	2747.98	99.4	—
Years	2010–2015	39	112,620	36,082	27.28 (18.14–38.85)	13435.38	99.7	0.1344
	2016–2020	26	118,979	53,521	32.14 (21.83–44.53)	7622.00	99.7	—
	2021–2025	6	70,939	9298	9.04 (1.46–39.94)	10011.52	100.0	—
Quality level	Low	4	2873	416	5.00 (0.14–67.06)	159.58	98.1	0.0009 ^∗∗∗^
	Medium	62	315,246	55,771	25.25 (18.32–33.73)	50130.79	99.9	—
	High	7	93,811	43,948	42.13 (34.36–50.32)	931.14	99.4	—
Background	Piglet	13	16,035	7483	28.54 (13.65–50.24)	1950.17	99.4	0.2384
	Weaning pig	9	6271	2243	31.71 (20.36–45.75)	628.81	98.7	—
	Fattening pig	14	14,138	6266	34.79 (22.57–49.41)	1443.41	99.1	—
	Gilt	5	13,256	5496	43.12 (35.38–51.20)	63.16	93.7	—
	Sow	15	31,818	14,316	29.44 (22.57–49.21)	607.16	97.7	—
	Boar	10	13,215	5751	41.24 (24.17–60.72)	522.49	98.3	—

^∗^indicates a statistically significant difference between the two groups. “ ^∗^”: *p* < 0.05; “ ^∗∗^”: *p* < 0.02; “ ^∗∗∗^”: *p* < 0.001.

**Table 4:** Estimated pooled prevalence of SIV across Chinese provinces (2010–2025).

Province	No. of studies	No. of sample	No. of positive	Prevalence with 95% CI (%)
Anhui	5	4029	1727	41.06 (28.06–55.44)
Chongqing	3	1455	804	49.93 (8.23–91.72)
Fujian	4	4422	2108	52.50 (29.40–74.58)
Gansu	3	1114	385	40.51 (22.56–61.42)
Guangdong	24	60,524	18,024	32.01 (20.99–45.48)
Guangxi	9	7087	1666	25.70 (12.41–45.77)
Guizhou	2	76	50	65.79 (54.49–75.54)
Hebei	5	3346	1354	39.43 (22.39–59.50)
Heilongjiang	1	377	213	56.50 (51.44–61.42)
Henan	8	3550	1055	34.97 (11.72–68.55)
Hubei	3	1293	721	24.93 (1.74–86.20)
Hunan	6	19,343	1330	46.13 (10.18–86.61)
Inner Mongolia	3	866	385	45.19 (16.64–77.30)
Jiangsu	4	5453	678	18.25 (4.36–52.22)
Jiangxi	5	5752	2768	47.22 (31.27–63.77)
Jilin	3	1559	255	22.83 (1.25–87.32)
Liaoning	7	51,176	8245	35.69 (17.10–59.90)
Ningxia	3	6621	1519	23.29 (6.18–58.33)
Shaanxi	2	634	54	7.56 (0–100)
Shandong	9	50,439	22,497	18.51 (4.96–52.22)
Shanghai	3	3554	1089	8.41 (0–99.64)
Shaanxi	4	1567	144	16.84 (1.41–74.07)
Sichuan	4	21,582	9734	56.84 (16.97–89.46)
Tibet	4	1869	942	54.27 (29.42–77.16)
Xinjiang	1	1519	553	36.41 (34.02–38.86)
Yunnan	1	322	120	37.27 (32.15–42.68)
Zhejiang	1	150	28	18.67 (13.21–25.71)

### 3.4. Sensitivity Analysis and Publication Bias

**Figure 4:**
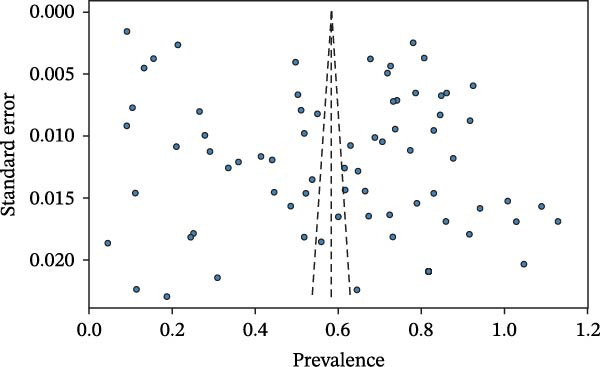
Funnel plot for the assessment of publication bias in the meta‐analysis of SIV prevalence. Each circle represents an individual study. The *y*‐axis represents the standard error of the prevalence, while the *x*‐axis shows the arcsine‐transformed prevalence. The dashed vertical line indicates the pooled effect size calculated using the random‐effects model. The dashed diagonal lines represent the pseudo 95% confidence interval limits. In the absence of publication bias, the studies are expected to be symmetrically distributed in an inverted funnel shape.

A leave‐one‐out sensitivity analysis confirmed the robustness of our pooled estimate. Sequentially removing each study resulted in recalculated pooled estimates (on the transformed scale) that remained within a narrow range (0.5758–0.5908), none of which deviated substantively from the original estimate of 0.5833 (Table 5). This demonstrates that our overall finding is not driven by any single influential study. Publication bias was evaluated using a funnel plot and Egger’s regression test. The funnel plot (Figure 4) was visibly asymmetric, with a notable lack of small‐sample studies reporting low prevalence in the bottom‐left quadrant. While high heterogeneity can contribute to such asymmetry, this pattern is often indicative of publication bias, where studies with nonsignificant or negative findings are less likely to be published. The Egger’s test provided quantitative support for this observation, confirming the presence of significant funnel plot asymmetry (*p* = 0.0009, data not shown). This finding suggests that publication bias may have inflated the overall pooled prevalence estimate for SIV in China, a critical limitation that must be acknowledged in the interpretation of our results.

**Table 5:** Results of the “leave‐one‐out” sensitivity analysis.

Excluded study No.	Recalculated pooled prevalence^a^	95% CI lower bound^a^	95% CI upper bound^a^
1	0.5877	0.5227	0.6528
2	0.5842	0.5186	0.6499
3	0.5805	0.5151	0.6459
4	0.5842	0.5186	0.6498
5	0.5876	0.5225	0.6527
6	0.5827	0.5171	0.6483
7	0.5885	0.5236	0.6533
8	0.5842	0.5186	0.6498
9	0.5795	0.5143	0.6447
10	0.5805	0.5151	0.6459
11	0.5900	0.5257	0.6543
12	0.5771	0.5127	0.6416
13	0.5813	0.5158	0.6469
14	0.5871	0.5219	0.6523
15	0.5852	0.5197	0.6508
16	0.5793	0.5141	0.6444
17	0.5806	0.5152	0.6460
18	0.5807	0.5153	0.6461
19	0.5902	0.5260	0.6544
20	0.5783	0.5135	0.6432
21	0.5888	0.5241	0.6535
22	0.5902	0.5260	0.6544
23	0.5787	0.5137	0.6437
24	0.5898	0.5255	0.6542
25	0.5812	0.5157	0.6467
26	0.5845	0.5189	0.6501
27	0.5787	0.5137	0.6437
28	0.5814	0.5158	0.6469
29	0.5813	0.5158	0.6468
30	0.5829	0.5172	0.6485
31	0.5843	0.5187	0.6500
32	0.5821	0.5165	0.6477
33	0.5822	0.5166	0.6478
34	0.5795	0.5143	0.6447
35	0.5824	0.5168	0.6481
36	0.5801	0.5148	0.6454
37	0.5763	0.5122	0.6404
38	0.5837	0.5180	0.6493
39	0.5899	0.5255	0.6542
40	0.5893	0.5247	0.6539
41	0.5825	0.5169	0.6481
42	0.5847	0.5191	0.6503
43	0.5814	0.5159	0.6470
44	0.5857	0.5202	0.6512
45	0.5758	0.5119	0.6397
46	0.5797	0.5144	0.6449
47	0.5820	0.5164	0.6476
48	0.5796	0.5144	0.6449
49	0.5896	0.5251	0.6540
50	0.5819	0.5163	0.6474
51	0.5853	0.5198	0.6508
52	0.5908	0.5268	0.6547
53	0.5829	0.5172	0.6485
54	0.5831	0.5175	0.6487
55	0.5871	0.5219	0.6523
56	0.5879	0.5229	0.6529
57	0.5774	0.5128	0.6420
58	0.5816	0.5161	0.6472
59	0.5864	0.5211	0.6518
60	0.5786	0.5136	0.6435
61	0.5838	0.5182	0.6494
62	0.5885	0.5237	0.6533
63	0.5802	0.5149	0.6456
64	0.5811	0.5156	0.6466
65	0.5868	0.5215	0.6521
66	0.5874	0.5222	0.6525
67	0.5813	0.5157	0.6468
68	0.5880	0.5230	0.6530
69	0.5840	0.5183	0.6496
70	0.5769	0.5125	0.6413
71	0.5799	0.5146	0.6452
72	0.5799	0.5146	0.6452
73	0.5844	0.5188	0.6500
Original pooled value	0.5833	0.5186	0.6481

*Note*: ^a^The values are on the arcsine‐transformed scale and are used to assess the stability of the results.

## 4. Discussion

Synthesizing data from 73 studies and over 410,000 samples, this meta‐analysis establishes a pooled prevalence of SIV in China of 30.3% (95% CI: 24.5%–36.4%) between 2010 and 2025. This figure confirms the endemic nature of SIV within China’s vast swine industry. Crucially, the extreme statistical heterogeneity (*I* ^2^ = 100%) underscores that this single value is an average across diverse epidemiological landscapes, shaped by geography, diagnostic approaches, viral genetics, and study quality. This discussion situates our findings within the global context, dissects the drivers of this heterogeneity, addresses the study’s limitations, and proposes a strategic path forward for surveillance and research.

Our 30.3% prevalence estimate serves as a vital national benchmark. Contextualizing this figure globally, a prior meta‐analysis reported a worldwide swine seroprevalence of 49.9%, with a specific estimate for Asia of 38.5% [102]. Our subgroup estimate for ELISA‐based seroprevalence (37.1%) remarkably mirrors this regional figure, lending credence to the external validity of our results and affirming that SIV circulates endemically in Asian swine herds. The higher global average reported by Baudon et al. [102] is likely attributable to their inclusion of more serological data from historically high‐prevalence regions and our study’s integration of virological data, which inherently capture the much lower prevalence of active infection (e.g., 2.6% by RT‐PCR). A review focused on extensively raised pigs reported a lower global seroprevalence (18.3%) and virological prevalence (1.3%) [103]. Our slightly higher virological rate may reflect China’s complex mosaic of farming systems, where intensive and backyard operations often coexist, potentially facilitating viral transmission. These comparisons underscore a critical message: meaningful interpretation of SIV prevalence demands careful consideration of the diagnostic, geographic, and systemic context.

The profound heterogeneity observed is a testament to SIV’s complex ecology, and our subgroup analyses successfully identified its principal drivers. The starkest driver was diagnostic methodology (*p*  < 0.0001), with an order‐of‐magnitude gap between seroprevalence (ELISA: 37.1%; HI: 32.9%) and virological prevalence (RT‐PCR: 2.6%; virus isolation: 0.9%). This is biologically expected: serology provides a cumulative record of lifetime exposure, while virology offers only a snapshot of active viral shedding during a brief infectious window (typically 5–7 days) [104]. This distinction is paramount for public health and veterinary policy, as conflating these two measures can lead to fundamentally flawed conclusions. Geographic location was another significant modulator (*p* = 0.0238), with Southwest China showing the highest prevalence (54.3%). This finding may be multifactorial, reflecting the region’s high pig density, extensive live‐animal trade networks, and a climate conducive to virus persistence, aligning with genomic studies that position China as a critical node in global SIV transmission networks [105]. Viral genetics also played a key role (*p* < 0.0001), confirming the dominance of H1N1 lineages (particularly EA H1N1) over H3N2 subtypes. This aligns with national‐scale molecular surveillance that identified EA H1N1 as the predominant lineage, highlighting its propensity for reassortment and zoonotic potential [17]. The emergence of specific genotypes like G4 EA H1N1, as reported locally in Shandong [10], exemplifies this rapid evolution.

While its reported isolation rate was low (0.47%), the contrast with our high pooled H1N1 seroprevalence illustrates how dominant strains dynamically evolve. It reveals a critical gap in our understanding of the national‐level circulation of new reassortants. Finally, we observed that high‐quality studies reported a significantly higher prevalence (42.1%) than their lower‐quality counterparts (*p* = 0.0009). This seemingly counterintuitive result likely reflects superior study design; high‐quality studies employing rigorous probability sampling are better powered to detect the true, often high, force of infection in endemic settings. The foremost strength of this study is its comprehensive scope, offering the most robust quantitative synthesis of SIV prevalence in China to date. Our bilingual search strategy minimized language bias, and extensive subgroup analyses provided crucial insights into the observed heterogeneity. However, several limitations must be acknowledged. First, we detected significant publication bias (Egger’s test, *p* = 0.0009), suggesting that studies reporting higher prevalence are preferentially published. Consequently, our 30.3% estimate may represent an overestimation of the true national prevalence. This highlights the need for prospective, multicenter surveillance programs and data‐sharing platforms that value null or negative findings. Second, to ensure the robustness of point estimates, we excluded studies with sample sizes below 427. While this threshold mitigated the impact of small‐study effects and outliers frequently linked to opportunistic sampling, we recognize that it may have omitted smaller, localized investigations offering granular insights into specific outbreaks. Third, a pivotal challenge in serology surveillance is the differentiation between naturally acquired and vaccine‐induced immunity. Despite our efforts to prioritize data from natural infections, the extensive use of SIV vaccines in China and inconsistent vaccination reporting in primary literature suggest that some ELISA/HI seropositivity may reflect vaccine‐derived antibodies, potentially leading to an overestimation of seroprevalence. Finally, substantial residual heterogeneity persisted even after subgroup analysis, likely stemming from unmeasured confounders such as herd vaccination status, biosecurity levels, and detailed demographic structures.

In conclusion, this meta‐analysis provides essential evidence to inform and refine SIV control strategies in China. To advance our understanding and mitigation efforts, future research must move toward: (1) integrated surveillance, combining virological, serological, and genomic approaches to create a holistic picture of infection dynamics and viral evolution; (2) longitudinal cohort studies, to precisely define subtype‐specific infection kinetics, immunogenicity, and zoonotic risk; and (3) spatio‐temporal modeling, to identify and quantify the environmental and anthropogenic drivers of SIV’s geographic patterns, enabling targeted interventions.

## 5. Conclusions

This large‐scale meta‐analysis, synthesizing 73 studies and over 410,000 samples, establishes that SIV is endemic in China, with a national pooled prevalence of 30.3% (95% CI: 24.5%–36.4%) from 2010 to 2025. This headline figure, however, masks a highly complex and heterogeneous epidemiological landscape (*I* ^2^ = 100%), which is significantly shaped by diagnostic methodology, geographical location, and viral subtype. While our overall estimate is methodologically robust, the detection of significant publication bias suggests the true national prevalence may be modestly lower than our estimate. Ultimately, this work confirms the substantial and persistent burden of SIV in China’s swine industry. The detailed prevalence estimates and the identified drivers of heterogeneity provide a crucial evidence base for refining risk assessments, designing targeted surveillance programs, and developing more effective, data‐driven control strategies.

**Nomenclature**
SIV: Swine influenza virus
IAV: Influenza A virus
HI: Hemagglutination inhibition
RT‐PCR: Real‐time reverse transcription polymerase chain reaction
ELISA: Enzyme‐linked immunosorbent assay
PRISMA: Preferred Reporting Items for Systematic Reviews and Meta‐Analyses
NOS: Newcastle–Ottawa Scale
REML: Restricted maximum likelihood
GLMM: Generalized linear mixed model
CI: Confidence interval.

## Author Contributions

Pengfei Zhao and Junlong Zhao conceived the study and designed the experiments. Xiutao Yang, Zhaofang Xi, and Qingxia Gao performed the data analysis and wrote the initial draft of the manuscript. Xiutao Yang, Zhaofang Xi, Qingxia Gao, and Pengfei Zhao conducted the literature search. Junlong Zhao and Zhaofang Xi supervised the project. Pengfei Zhao was responsible for project guidance and manuscript revision.

## Funding

This research was financially supported by the Fundamental Research Funds for the Central Universities in China (Grant 2662020DKPY016).

## Disclosure

All authors have read and approved the final manuscript for publication.

## Conflicts of Interest

The authors declare no conflicts of interest.

## Supporting Information

Additional supporting information can be found online in the Supporting Information section.

## Supporting information


**Supporting Information** Table S1 provides a comprehensive bibliographic list of the 73 primary studies included in this systematic review and meta‐analysis, detailing the article titles, first authors, publication years, and methodological quality assessment scores for each study.

## Data Availability

The data that support the findings of this study are available from the corresponding author upon reasonable request.
