# Supplementary material for: Prevalence and Heterogeneity of Swine Influenza Virus in China From 2010 to 2025: A Systematic Review and Meta‐Analysis
Source: Transbound Emerg Dis. 2026 Feb 13;2026:1096796. doi: 10.1155/tbed/1096796 (PMC12904846; doi:10.1155/tbed/1096796)
Supplement: Supplementary file 1 — Supporting Information Table S1 provides a comprehensive bibliographic list of the 73 primary studies included in this systematic review and meta‐analysis, detailing the article titles, first authors, publication years, and methodological quality assessment scores for each study. [file TBED-2026-1096796-s001.docx]

Supplementary Table S1. List of 73 studies included in the meta-analysis

| No. | Article Title | First Author | Year | Score |
| --- | --- | --- | --- | --- |
| 1 | Prospective surveillance for influenza A virus in Chinese swine farms | Anderson BD | 2018 | 7 |
| 2 | Molecular evolution of H1N1 swine influenza in Guangdong, China, 2016–2017 | 1. K. Cai | 2018 | 7 |
| 3 | Serological investigation and analysis of swine influenza virus infection in intensive pig farms in parts of Guangdong Province | 1. P. Cao | 2013 | 7 |
| 4 | Serological survey of H1N1 and H3N2 subtypes of swine influenza virus in Guangxi from 2009 to 2013 | Y Chen | 2015 | 7 |
| 5 | Long-term co-circulation of multiple influenza A viruses in pigs, Guangxi, China | 1. Q. Huang | 2024 | 5 |
| 6 | Serological investigation and analysis of swine influenza in Liaoning province from 2012 to 2017 | 1. S. Lan | 2019 | 6 |
| 7 | Prevalence, Genetics and Evolutionary Properties of Eurasian Avian-like H1N1 Swine Influenza Viruses in Liaoning | 1. L. Li | 2022 | 6 |
| 8 | Serological evidence of hepatitis E virus and influenza A virus infection in farmed wild boars in China | 1. L. Liang | 2019 | 8 |
| 9 | Serological survey of swine influenza in major pig-rearing provinces of China from 2012 to 2013 | 1. P. Liu | 2014 | 6 |
| 10 | Investigation of serological antibodies against influenza virus in swine populations in some provinces of China | 1. Y. Sui | 2016 | 5 |
| 11 | Genome-scale evolution and phylodynamics of swine influenza A viruses in China: a genomic epidemiology study | 1. L. Sun | 2025 | 5 |
| 12 | Serological survey of H1 and H3 antibodies of swine influenza in Fujian province | 1. B. Wang | 2011 | 6 |
| 13 | Serological monitoring of swine influenza in different regions of China from 2011 to 2012 | H Yin | 2013 | 6 |
| 14 | Serological survey of H1N2 subtype of swine influenza virus in Guangxi | 1. X. Zhang | 2011 | 5 |
| 15 | Avian-like A (H1N1) swine influenza virus antibodies among swine farm residents and pigs in southern China | H Zhou | 2014 | 4 |
| 16 | Serological survey of swine influenza and H1N1/2009 pandemic influenza virus in pig farms in South China | 1. Y. He | 2015 | 7 |
| 17 | Serological Surveillance of the H1N1 and H3N2 Swine Influenza A Virus in Chinese Swine between 2016 and 2021 | 1. Z. Zhao | 2022 | 10 |
| 18 | Serological survey of H1 and H3 subtype influenza viruses in swine populations in Jiangxi Province from 2012 to 2015 | P Gan | 2017 | 5 |
| 19 | Continued evolution of the Eurasian avian-like H1N1 swine influenza viruses in China | F Meng | 2023 | 7 |
| 20 | Identification of an H1N1 subtype of swine influenza virus and serological analysis | 1. C. Sun | 2019 | 5 |
| 21 | Identification of an H6N6 swine influenza virus in southern China | 1. H. Zhang | 2011 | 4 |
| 22 | Two genotypes of H3N2 swine influenza viruses identified in pigs from Shandong Province, China | 1. Z. Zhao | 2024 | 5 |
| 23 | Etiological and serological investigation of swine influenza virus in swine populations in South China | X. L. Fu | 2020 | 5 |
| 24 | Genetic evolution analysis of avian-like H1N1 swine influenza virus in Shaanxi Province | 1. J Ren | 2013 | 5 |
| 25 | Antibody detection of H1 subtype swine influenza and Streptococcus suis type 2 in clinical isolated sera | C Wu | 2017 | 5 |
| 26 | Monitoring and analysis of H1N1 subtype swine influenza in Dongguan City from 2009 to 2018 | 1. Q. Cai | 2020 | 7 |
| 27 | Serological survey of H1 and H3 subtype swine influenza in some pig farms | 1. C. Chen | 2012 | 6 |
| 28 | Serological investigation of H1N1 subtype swine influenza antibodies in Dongguan city | 1. W. Chen | 2018 | 5 |
| 29 | Epidemiological investigation of swine influenza in Fujian province and genome sequence analysis of A/Swine/Fujian/F1/2010 (H1N2) strain | 1. H. Yu | 2012 | 7 |
| 30 | Seroepidemiological survey of swine influenza (H1N1 subtype) in intensive pig farms in Shandong province | 1. Z. Huang | 2014 | 6 |
| 31 | Epidemiological investigation of swine influenza in Ningxia slaughterhouses | 1. X. Li | 2012 | 5 |
| 32 | Investigation on the current status of swine influenza in intensive pig farms in Ningxia | 1. X. Li | 2013 | 6 |
| 33 | Serological survey of Tibetan swine influenza virus along the Sichuan-Tibet Highway in Maizhokunggar and Gongbo counties | X. B. Ma | 2015 | 6 |
| 34 | Serological survey of H3N2 subtype swine influenza in Dongguan city from 2015 to 2017 | 1. Q. Ouyang | 2018 | 6 |
| 35 | Serological monitoring and analysis of mammalian influenza viruses in Xinjiang | P Wang | 2013 | 6 |
| 36 | Seroepidemiological survey of swine influenza in intensive farms in Liaoning province from 2019 to 2022 | 1. Y. Wei | 2023 | 7 |
| 37 | Serological survey of H1 and H3 subtype swine influenza virus antibodies in Guangdong province | M Xu | 2010 | 5 |
| 38 | Serological survey of swine influenza in slaughterhouses in parts of China in 2016 | Q Xu | 2018 | 6 |
| 39 | Molecular epidemiological study of swine influenza in Shanxi province | 1. M. Yao | 2012 | 7 |
| 40 | Epidemiological investigation of H1N1 swine influenza in intensive pig farms in Hunan province | 1. Y. Yu | 2011 | 7 |
| 41 | Investigation on the prevalence of novel swine-origin influenza A virus in pig farms from 2010 to 2011 | 1. N. Zhang | 2013 | 4 |
| 42 | Investigation of swine influenza in intensive pig farms in the border area of Hebei, Shandong, and Henan provinces | 1. G. Cai | 2016 | 7 |
| 43 | Epidemiological investigation of SIV in South China and study on differential lung expression profiles of mice infected with GDK6 mouse-adapted strain | 1. P. Cao | 2017 | 10 |
| 44 | Study on genetic evolution and pathogenicity of H1 subtype swine influenza virus from 2020 to 2021 | X. X. Cui | 2022 | 6 |
| 45 | Serological investigation and analysis of swine influenza in intensive pig farms | 1. Q. Zhai | 2012 | 5 |
| 46 | Epidemiological investigation of SIV in South China and study on the mechanism of ammonium glycyrrhizinate against influenza virus | B Fang | 2018 | 5 |
| 47 | Isolation, identification and serological investigation of swine influenza virus in Shandong province from 2020 to 2021 | 1. B. Han | 2022 | 8 |
| 48 | Serological investigation of swine influenza virus infection in Central South China from 2017 to 2020 | X. K. Hu | 2023 | 6 |
| 49 | Epidemiological investigation and biological characteristics of isolated strains of swine influenza in China from 2022 to 2023 | 1. L. Huang | 2024 | 5 |
| 50 | Antibody monitoring and virus isolation of H1 and H3 subtype swine influenza in parts of Guangdong province | 1. Z. Huang | 2011 | 6 |
| 51 | Serological investigation and analysis of H3N2 influenza A in swine populations in a district of Shanghai city | 1. Y. Jin | 2014 | 5 |
| 52 | Investigation of major viral infections in commercial pigs in Shanghai slaughterhouses from 2011 to 2014 | 1. B. Ju | 2015 | 3 |
| 53 | Serological survey of H1N1 subtype swine influenza in Cangzhou area | 1. M. Liu | 2015 | 5 |
| 54 | Statistical analysis of serological study of swine influenza in Gansu province | 1. Q. Liu | 2014 | 6 |
| 55 | Serological survey of H1N2 subtype swine influenza virus and study on immunization effect in Guangxi | 1. R. Lu | 2011 | 6 |
| 56 | Serological survey of H1 subtype swine influenza virus infection | X. S. Luo | 2011 | 5 |
| 57 | Serological investigation and analysis of swine influenza in intensive pig farms in Chongming area in the past three years | 1. P. Shi | 2014 | 5 |
| 58 | Serological survey of swine influenza and H1N1/2009 pandemic influenza virus in pig farms in Anhui area | 1. Y. Wang | 2016 | 8 |
| 59 | Serological survey of swine influenza in Ningxia province | 1. X. Wang | 2012 | 5 |
| 60 | Serological survey of three subtypes of swine influenza in Shandong province | 1. C. Wang | 2018 | 7 |
| 61 | Serological investigation and analysis of H3N2 subtype swine influenza in Dongguan city in the past 5 years | 1. Y. Xie | 2017 | 7 |
| 62 | Analysis of genetic evolution, receptor characteristics and pathogenicity of swine influenza virus in Guangdong province from 2017 to 2019 | 1. N. Yu | 2020 | 7 |
| 63 | Isolation, identification and serological investigation of swine influenza virus from 2018 to 2020 | 1. Z. Zhao | 2021 | 8 |
| 64 | Serological survey of swine influenza virus and study on the effect of HA protein cleavage site changes on the pathogenicity of H1N2 SIV | 1. T. Zhong | 2020 | 8 |
| 65 | Epidemiological investigation of H1N1 and H3N2 subtypes of swine influenza virus in Nanjing area | X. L. Zhu | 2012 | 7 |
| 66 | Isolation, identification and biological characteristics analysis of swine influenza virus in Guangdong province from 2016 to 2017 | X. H. Zhu | 2019 | 6 |
| 67 | Seroepidemiological survey of swine influenza in Liaoning area | Y Gao | 2019 | 7 |
| 68 | Seroepidemiological survey of influenza A, hepatitis E, mycoplasma pneumonia, and Haemophilus parasuis in wild boars | 1. L. Liang | 2019 | 7 |
| 69 | Monitoring and analysis of swine influenza antibodies in some pig farms in Guangdong province | W Lin | 2012 | 6 |
| 70 | Preliminary report on epidemiological investigation of H1 and H3 subtypes of swine influenza in Tibetan pigs in Nyingchi, Tibet | 1. B. Luo | 2016 | 6 |
| 71 | Investigation of H5N6 subtype influenza virus in pig farms in Guangdong province and study on pathogenicity of an H1N1 SIV strain in mice | F Qin | 2017 | 6 |
| 72 | Investigation of swine influenza virus in intensive pig farms in Anyang city from 2018 to 2019 | 1. Y. Wang | 2021 | 6 |
| 73 | Monitoring and risk assessment of swine influenza in Dongguan city | 1. Y. Xie | 2012 | 6 |
